# A Deep Dive of Autoencoder Models on Low-Contrast Aquatic Images

**DOI:** 10.3390/s21154966

**Published:** 2021-07-21

**Authors:** Rich C. Lee, Ing-Yi Chen

**Affiliations:** Department of Computer Science and Information Engineering, National Taipei University of Technology, Taipei 23741, Taiwan; ichen@ntut.edu.tw

**Keywords:** autoencoder, deep learning, computer vision, image recognition

## Abstract

Public aquariums and similar institutions often use video as a method to monitor the behavior, health, and status of aquatic organisms in their environments. These video footages take up a sizeable amount of space and require the use of autoencoders to reduce their file size for efficient storage. The autoencoder neural network is an emerging technique which uses the extracted latent space from an input source to reduce the image size for storage, and then reconstructs the source within an acceptable loss range for use. To meet an aquarium’s practical needs, the autoencoder must have easily maintainable codes, low power consumption, be easily adoptable, and not require a substantial amount of memory use or processing power. Conventional configurations of autoencoders often provide results that perform beyond an aquarium’s needs at the cost of being too complex for their architecture to handle, while few take low-contrast sources into consideration. Thus, in this instance, “keeping it simple” would be the ideal approach to the autoencoder’s model design. This paper proposes a practical approach catered to an aquarium’s specific needs through the configuration of autoencoder parameters. It first explores the differences between the two of the most widely applied autoencoder approaches, Multilayer Perceptron (MLP) and Convolution Neural Networks (CNN), to identify the most appropriate approach. The paper concludes that while both approaches (with proper configurations and image preprocessing) can reduce the dimensionality and reduce visual noise of the low-contrast images gathered from aquatic video footage, the CNN approach is more suitable for an aquarium’s architecture. As an unexpected finding of the experiments conducted, the paper also discovered that by manipulating the formula for the MLP approach, the autoencoder could generate a denoised differential image that contains sharper and more desirable visual information to an aquarium’s operation. Lastly, the paper has found that proper image preprocessing prior to the application of the autoencoder led to better model convergence and prediction results, as demonstrated both visually and numerically in the experiment. The paper concludes that by combining the denoising effect of MLP, CNN’s ability to manage memory consumption, and proper image preprocessing, the specific practical needs of an aquarium can be adeptly fulfilled.

## 1. Introduction

A common approach to tracking aquatic subjects in an aquarium is to capture their movements through a video camera [1]. The challenge here is not just about recording these videos over an extended period, but also the extraction of the images from the footage. Many neural network architectures have demonstrated the capability to identify objects from within specific sample images [2]. When applying these pre-trained models on specific targets in various environments, not only does the neural network structures need to be changed often, but a series of image preprocessing is also required to provide a more precise model [3,4].

The identification of a subject in its murky surroundings from the footage is often a challenge to the pre-trained model prediction process. The results may vary due to several uncertain and inevitable factors: (1) the subjects’ captured motions may not be as clear as those in the training dataset [5]; (2) the condition of the water in the collected footage may not be as clean as those in the training dataset [6]; and (3) changes in the light source and changes in amount of light may cause too much ambiguity to identify the subjects with certainty [7]. These uncertainties highlight the importance of image preprocessing in the neural network model training and prediction.

Once the subjects are identified by the trained model, the next challenge is to store these images into a repository for further analysis [8]. Since these images were captured in an ever-changing the environment, such as the cycle between clear and cloudy water after water changing procedures [9], storing these images in their source format is ineffective. This is because: (1) to capture the source images well, it usually requires high resolution and dimension video devices; (2) the captured conditions are often inevitably mixed with visual noise; and (3) storing these source images over time requires an enormous amount of disk space and is thus costly.

The autoencoder is a special application of deep learning; it aims to reduce the dimensionality of the image and generates a short binary representation, the latent space of the image, so that it can be reconstructed back within an acceptable loss range in comparison to the source. This paper argues that this latent space can be effectively used and stored into a repository, a must for an aquarium’s routine operations. It also systematically discusses the associated impacts of the various autoencoder configurations when applied to low-contrast images of aquatic beings.

This paper seeks to find a practical approach to the use of autoencoders in the context of serving an aquarium’s needs. The goal is to reduce the dimensionality of the growing collection of the aquatic subjects over time, to store these videos effectively and efficiently, and to retain useful and desirable visual information. In pursuit of this goal, the paper answers several questions regarding the outcome and impact of applying the autoencoder technique: (1) what are the key differences between the Multilayer Perceptron (MLP) and Convolution Neural Networks (CNN) autoencoder in terms of the model architecture and their associated parameters; (2) how do autoencoder model parameters impact the outcome; (3) can autoencoder techniques effectively reduce repository spaces; (4) can those regenerated images from the autoencoder adequately serve aquarium research and management purposes; (5) can autoencoder models enhance the collected low-contrast images; and (6) what are the necessary steps and preparations required for an aquarium to apply the proposed autoencoder framework of this paper.

## 2. Related Works

The autoencoder has a symmetric neural network structure: the encoder, and the decoder. The encoder layer extracts the essence from the input images, while the decoder layer uses that extracted essence to reconstruct the image. Both layers are trying to minimize loss and converge into the trained model. It has various applications, such as: (1) dimensionality reduction—to reduce the high dimensional images into low binary dimensional latent space [10]; (2) feature extraction—since the latent space can be reconstructed back to the source, they are considered the most important features of the image [11]; (3) image compression—applying the convolutional networks to construct the autoencoder is a common way of compressing the source image [12]; (4) image denoising—by adding random noises into the source image and letting the autoencoder model train a denoise model, it can then reconstruct noisy images back to a form similar to the source [13]; and (5) image generation—whether the learned latent space can be reconstructed to resemble the source will depend on the quality of the input images [14], etc.

Both MLP and CNN autoencoder approaches were widely applied by many previous works, but their input data dimensions were much less than the collected images by this paper, hence the preprocessing and the model design require special attention. Table 1 illustrates the comparison between the previous works and this paper.

The previous work by Ji et al. [15] applied an MLP framework against two famous image datasets, MNIST—the monochrome image dimension is $\left(28,\;28,\;1\right)$, and CIFAR10—the color image dimension is $\left(32,\;32,\;3\right)$; both input image dimensions in their autoencoder models were smaller and less complex than the low-contrast images used in this paper. Thus, their autoencoder models are not suitable for the purpose of this paper.

A medical study on HIV classification proposed the measurement of the image difference between the source and the reconstructed image, and the standardization function [16] is similar to this paper’s Equation (4). This paper applied the MLP framework to denoise the low-contrast source images by generating the standardized difference images. Wang et al. [17] applied the CNN autoencoder similar framework as a part of the image preprocesses to extract the essence from the source images.

The work of Pihlgren et al. [18] discussed the improvement of the image autoencoder by applying the perceptual loss function to compare the images from high-level representation perspectives. The work applied the CNN framework, a model structure similar to that of this paper, with the difference being that higher contrast input source images were used, with smaller latent spaces, and lower image dimensions—$\left(64,\;64,\;3\right)$ and $\left(96,\;96,\;3\right)$—than those used in this paper. Since the source images used in this paper are low contrast, the autoencoder cannot extract the features to generate the adequate latent space in small size and reconstruct the source image with less loss.

In the work of Dumas et al., a way was proposed to minimize the rate-distortion optimization: by giving the optimal quantization step size, the compression outcome will be better [19]. Similarly, this paper applied a more intuitive approach—by enhancing the source images—to train the autoencoder and apply the same method to reconstruct the low-contrast images.

In Khan et al. [20] work, they applied two layers of one-dimensional convolution and then connect to the autoencoder to detect the misuses from the potential cyberattacks. Since the source datasets used in this paper were quite different than their work, certain image preprocessing must be connected before training the model; on the other hand, this paper discloses the effects between CNN and MLP approaches under various configurations, which makes the contribution unique.

The work of Ramalho et al. used natural language as the input source data to the autoencoder to get the latent representations and used these latent data to generate the corresponding synthetic 3D images [21]. This paper used the images from the aquatic live habitat as the input data; the denoising techniques are different than their work as well as the purpose of the research.

Low-contrast images are defined by the contours of the subjects being blurry in contrast to the background. It implies that the color slopes are slim, and thus difficult to find derivatives when the activation function is applied during the model training. However, if the contours of the subject are too blurry, it will blend into the background and be treated as a “blank” image, making it difficult to obtain a convergent training model.

For the purpose of this paper, several videos of fish were collected from an aquarium and were then converted into a sequence of images, with most of the frames being low-contrast. An object detection neural network model was then trained and used to predict the position of the detected subjects relative to the image. The sample images from the video frames were used as the source to extract the contours of the fish based on their relative coordinates.

## 3. Aquatic Subject Sample Image Dataset

**Figure 1:**
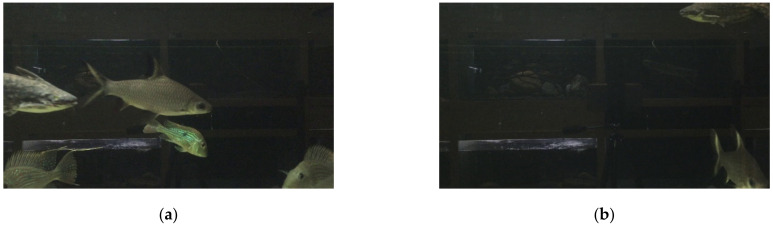
The sample aquatic subject frames, (**a**) multiple subjects and (**b**) low-contrast subjects in their frames.

The paper collected videos of aquatic subjects from the Aquatic Animal Center (National Taiwan Ocean University Aquatic Animal Center (http://aac.ntou.edu.tw/, accessed on 20 April 2021)). These videos contain various species of fish moving about over time. The low-contrast and high-noise images were randomly chosen to see if the autoencoder model can help in dimensionality reduction. The number of samples was approximately equal for each species. Figure 1 illustrates some sample frames of the subjects. Both captured images were from a video recording of the same compartment in the aquarium. There are other compartments behind the one in focus and can be seen through the murky water, which serves as visual noise. Of the two samples chosen, (a) shows multiple subjects swimming in front of background noise, while (b) only partially shows some of the subject in low contrast, with most of the image only containing visual noise. From these samples, they clearly showed there were noisy background (reflections, shades, and a metal bar), and the light illuminated from the left and fade to the right in the compartments. These noisy backgrounds will interfere with the outcomes of the autoencoder.

**Figure 2:**
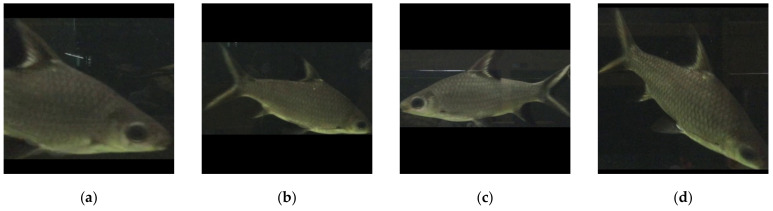
Sample standardized aquatic object images with various appearances: (**a**) partial body, (**b**) full body, (**c**) body with nose, and (**d**) diving body.

Since the aquatic objects are the focal points, not the noisy backgrounds, a following research question is raised: what would the autoencoder learn only from the aquatic objects and can this autoencoder reconstruct back to the full image? To prove that, this paper cropped the aquatic objects out of the source images and standardized them into a unified size (taking the maximum value from the object dimension as the square’s width), illustrated in Figure 2. The purpose of this squaring scheme is to prevent distortion and retain the subjects’ aspect ratios when the model training requires the same dimension for all input images. The paper posits that, if there were a way to reduce the background noises and reveal the aquatic object shapes well, so that the caregivers could observe their living condition conveniently. Since the size of the input images is smaller and the number of aquatic object samples is also more than the full image, the autoencoder might have better outcomes.

## 4. Autoencoder Framework

**Figure 3:**
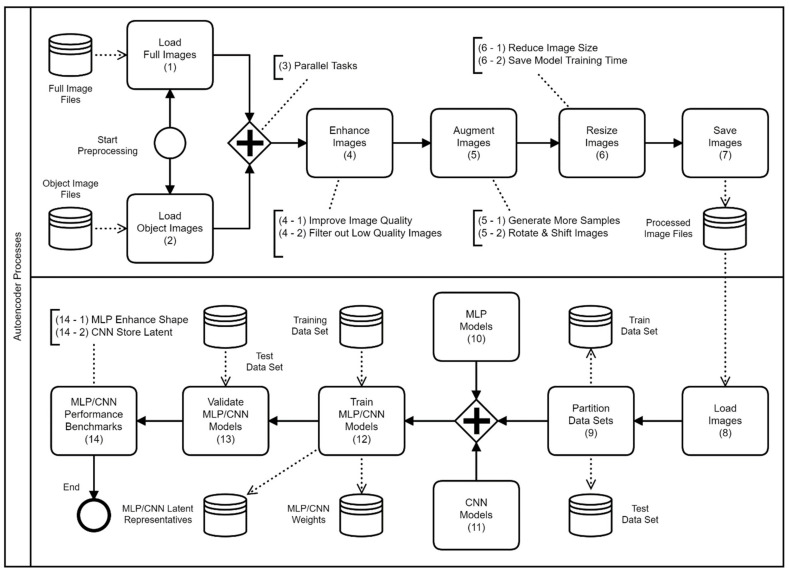
The proposed autoencoder framework.

The autoencoder models designed in this paper are derived from two widely applied approaches, namely MLP [16,22] and CNN [23,24]. The authors have shared the autoencoder codes on the GitHub (The GitHub link of the shard autoencoder programs is at https://github.com/rich58lee/autoencoder (accessed on 20 April 2021)). Figure 3 illustrates the proposed autoencoder framework consisting of two separate processes; the upper process is used to populate and preprocess the datasets, while the lower process is the actual autoencoder model training process. In the diagram below, the thin lined circle represents where the process starts, while the bold lined circle represents where the process ends. The rectangles represent the tasks, the cylinders represent a repository, database, or files, and the diamond shape with a plus sign represents two parallel process branches.

In the preprocess part, there are two branches of tasks: (1) “Load Full Images”—reads all full-image files into a high-dimensional matrix and proceeds the following diamond-plus tasks; (2) “Load Object Images”—reads the object image files extracted by an object detector; each object image contains only one object. The diamond-plus contains a series tasks of: (4) “Enhance Images”—filtering out those images with exceptionally low quality, smoothing the images with a filter kernel and amplifying their contrast; (5) “Augment Images”—to prevent model overfitting side-effect, populating more samples by shifting the coordinates and making rotations (in every 45°); this augmentation reflects the reality that the aquatic subjects may be present various ways in their habitat; (6) “Resize Images”—considering the practical use, decreasing the image size by applying an interpolation method to save the model training time; and (7) “Save Images”—storing these preprocessed images onto the repository.

In the model training part, the task (8), “Load Images”, loads the preprocessed images into a high-dimensional matrix from the repository, and then conducts the task (9), “Partition Data Sets”, to partition this matrix into two datasets (the ratio between the datasets of the train and the test is 80% and 20%) randomly. The diamond-plus parallel tasks are: (10) “MLP Models”—given various dimensions to formulate the model structures and conducting the training for each model; (11) “CNN Models”—by given the same dimensions as MLP to formulate several convolution networks, and then proceed with a series of neural network following tasks.

The task (12), “Train MLP/CNN Models”, begins the model training; it reads the image files from the “Training Data Set”, and then derives the model weights and the training history; the task (13), “Validate MLP/CNN Models”, applies the model against the “Test Data Set”; and finally, the task (14), “MLP/CNN Performance Benchmarks”, presents the reconstructed images visually.

When applying the autoencoder techniques, several interesting research questions need to be answered: (1) how do model configurations affect the outcome; (2) is the neural network structure better if it is more complex (3) how to achieve the optimal length of the latent space; and (4) will image preprocessing affect the training result?

## 5. Environment Setup

This paper used a high-performance computer equipped with: (1) Intel(R) Core(TM) i9-9900KF CPU @3.60 GHz; (2) RAM 128 GB; (3) GPU NVIDIA TU102 (GeForce RTX 2080 Ti); and (4) Xeon E3-1200 v5/E3-1500 PCIe for 2 TB SSD. This computer was running the Ubuntu operating system (the 20.04 LTS version); the CPU performance of generating 10,000 prime numbers took only 10 s to complete by the *sysbench*, a modular, cross-platform and multi-threaded benchmark utility.

## 6. Autoencoder Model Design

The input images were all placed into a square format with the dimensions of 256 × 256 px with the original ratio of the images preserved to save computing resources and training time. The remaining empty spaces were filled with zeros, resulting in the spaces being filled with black.

### 6.1. MLP Autoencoder Models

The baseline settings were: (1) the latent code length: 64; (2) the training epochs: 512; (3) the batch size: 8; (4) the activation functions: Rectified Linear Unit (ReLU) and Sigmoid [25]; (5) the optimization algorithm: Adam [26]; (6) the loss function: binary cross entropy [27]; and (7) the training model: based on fully connected layers (denoted as Dense), illustrated in Table 2.

The standardized function is illustrated in Equation (1): x is a pixel value, and X is the whole image matrix. The Equation (2) illustrates the image difference calculation for visualization; the purpose is to ensure that all pixel values will be within 0≤x≤1. Equation (3) calculates the RGB color (red, green, or blue) difference between the two images. A benchmark function (the total differences over the RGB color planes) is defined in Equation (4): simply take the square root of the summation of all color differences. The higher the benchmark value, the higher the visual contrast.

(1) $$standardization\left(X\right)= [x-min\left(X\right)]/ [max\left(X\right)-min\left(X\right)]$$

(2) $$image_{diff\left(X,Y\right)}=standardization\left(X-Y\right)$$

(3) $$Color_{diff}\left(X,Y,c\right)={\left\{X_{c}-Y_{c}\right\}}^{2}$$

(4) $$benchmark\left(X,Y\right)=\sqrt{\overset{2}{\underset{c=0}{\sum }}Color_{diff}\left(X,Y,c\right)}$$

**Figure 4:**
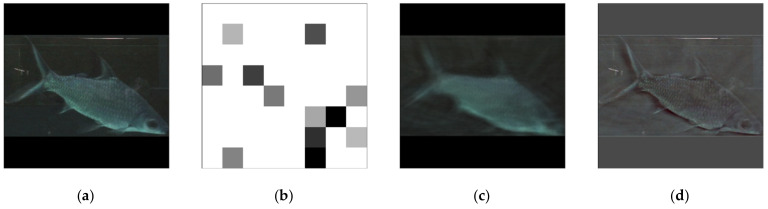
The MLP autoencoder sample results: (**a**) source, (**b**) latent, (**c**) reconstructed, and (**d**) difference.

The validation result, illustrated in Figure 4, consists of four parts: Figure 4a—the source image; Figure 4b—the standardized latent space; Figure 4c—the reconstructed image through the autoencoder; and Figure 4d—the standardized difference image between Figure 4a,c. The random sample’s benchmark value was 0.1779; it can be treated as the performance of the autoencoder. It is worth noting here that the difference image (Figure 4d) provides a much clearer visual of the subject’s body in comparison to the reconstruction. The model converged at the 151st epoch, the loss and the validation loss were (0.2640, 0.2695), respectively. The model extracted the body contour noise, which is why the standardized difference image (a subtraction between the source (Figure 4a) and the reconstructed image (Figure 4c)) shows a much clearer shape.

The next experiment was to add additional symmetric layers, namely IN→EN512→EN256, and DE256→DE512→OUT. The latent space length was $12^{2}$; it remained in a squared-value so that it can be visualized. The model converged at the 101st epoch; the loss and the validation loss were (0.2642, 0.2701), respectively. As expected, the standardized difference image again showed a clearer shape of the subject, this means the autoencoder has effectively filtered the noise out of the source images.

The last experiment is based on the previous model structure, but with enhanced source images before the model training. The image enhancement preprocess set the brightness factor to 2 $\left(valuerange:\;0\leq b\leq 100\right)$, the contrast factor to 0.6 $\left(valuerange:1.0\leq b\leq 3.0\right)$, and applied a (3, 3) filter ($\left\{ [-1,-1,-1],\;\; [-1,11,-1],\;\; [-1,-1,-1]\right\}$) to the source images. The model converged at the 147th epoch, with the loss and the validation loss being (0.3698, 0.3756), respectively. The sample low-contrast source images that were omitted during the initial model training process due to their exceptionally low quality, “unseen” by the autoencoder training model, so to speak, were then tested through the autoencoder, which was able to reconstruct the source and produce a benchmark value of 0.7301. This means the model has been effectively trained and could even work with images previously deemed too low quality for model training.

**Figure 5:**
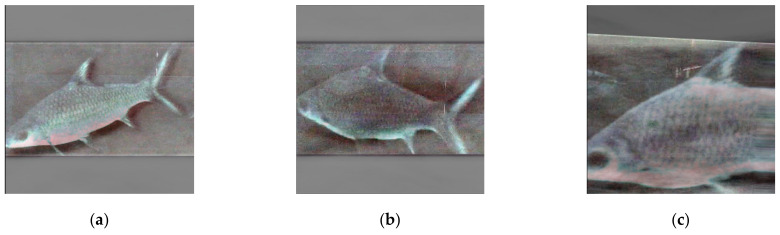
The image differences from various random samples: (**a**) difference from the test set, (**b**) difference from the “unseen” samples, and (**c**) difference from the “unseen” samples II.

This paper concluded that changing the MLP autoencoder’s configuration does not improve the reconstruction from a low-contrast source image. However, an unexpected finding was that the difference images (as illustrated in Figure 5), managed to preserve the subjects’ contours extremely well. Figure 5a was constructed from the test set, while Figure 5b,c were constructed from the “unseen” (omitted from the initial model training due to exceptionally low quality) low-contrast images. This implies the common features of the source images has been extracted as noise patterns during the autoencoding process. There were attempts to experiment with more complex MLP models, however these models were unable to be deployed due to requiring addition computing resources (beyond what would be considered practical for an aquarium). Since MLP could not reach the research goal, image convolution and pooling scheme (CNN) may be worth exploring.

### 6.2. CNN Autoencoder Models

The baseline settings were: (1) the latent space: (32, 32, 64); (2) the training epochs: 512; (3) the batch size: 8; (4) the activation functions: ReLU and Sigmoid; (5) the optimization algorithm: Adam; (6) the loss function: mean-square error (MSE); and (7) the training model: the encoding part consists of two blocks, each block contains two-dimensional convolution and the max pooling layers, and the decoding part is symmetrical to the encoding but built in reverse, as illustrated in Table 3. The convolution’s kernel size is (3, 3), while the max pooling’s pool size is (2, 2).

The CNN model applies two-dimensional convolutions against the input images and receives the maximum values out of the pooling matrices to encode the latent space. This derived latent space is then used to apply the reverse convolution transposition and the up-sampling process to reconstruct the source image. The model converged at the 365th epoch, the loss and the validation loss were (4.1545 × 10^−5^, 4.2398 × 10^−5^), respectively.

**Figure 6:**
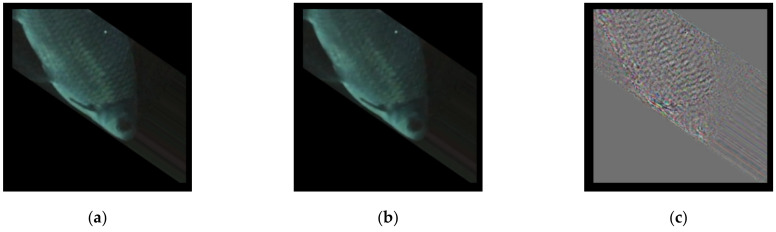
The CNN autoencoder sample results: (**a**) source, (**b**) reconstructed, and (**c**) difference.

The sample standardize difference image benchmark value (the total differences over the RGB color planes calculated by Equation (4)) was 0.2199. The standardized difference image showed that the contours were far less clear than MLP’s. The Figure 6 illustrates: Figure 6a—the source image, Figure 6b—the reconstructed image, and Figure 6c—the standardized difference image. Not surprisingly for this effect, CNN’s latent code contains more information than MLP’s, $256^{2}>8^{2}$.

The next experiment was to reduce the dimension spaces, CN_256→CN_128, CN_128→CN_64, and CN_64→CN_32, but the model structure remained as the baseline. The latent space thus reduced to (32, 32, 32). The standardized image difference was 0.29380453±0.021411419. The model converged at the 379th epoch; the loss and the validation loss were (4.5114 × 10^−5^, 4.3126 × 10^−5^), respectively. The sample image benchmark value was 0.03299. The result showed that reducing the dimension spaces improved the outcome (because the benchmark value 0.03299<0.2199) and consumed much less computing time in comparison with the baseline.

The following experiment aimed to reduce the dimension spaces further, CN_256→CN_64, CN_128→CN_32, and CN_64→CN_16, but with the model structure remaining as the baseline. The latent space thus reduced to $\left(32,\;32,\;16\right)$. The standardized image difference was 0.47967815±0.027717976. The model stopped at the 512th epoch because the losses had not improved since the previous intermediate result; the loss and the validation loss were (5.5957 × 10^−5^, 5.2107 × 10^−5^), respectively.

The sample difference image benchmark value was 0.4075, and when the model was applied to another “unseen” sample, an identical difference image benchmark value was generated, indicating that the model is stable. The result showed that by reducing the dimension spaces further, it had made the performance slightly worse (0.4075>0.2199). However, the autoencoder consumed much less computing time in comparison with the baseline.

Will image enhancement help with the autoencoder? The following experiment was to enhance the source image first by setting the brightness factor to 2 (valuerange: 0≤b≤100), the contrast factor to 0.6 (valuerange: 1.0≤b≤3.0), and a $\left(3,\;3\right)$ filter ($\left\{ [-1,-1,-1], [-1,9,-1], [-1,-1,-1]\right\}$). The model structure and the latent space remained as the same as the previous one. The model converged at the 420th epoch. The loss and the validation loss were (1.5203 × 10^−4^, 1.5547 × 10^−4^), respectively. The sample standardized difference image benchmark value was 0.1754. Applying the model to another “unseen” sample also got the identical standardized difference image benchmark value, indicating that the model is stable.

In comparison to the previous model, the image enhancement model has a better benchmark value (0.1754<0.4075), meaning the reconstructed image has a higher contrast than the unenhanced model. Furthermore, by taking the proper image enhancement treatment, the autoencoder still generated good results in terms of the outcome (the benchmark value 0.1754<0.2199) and the computing time consumption, in comparison to the baseline model.

Can the autoencoder learn from the full images and still reconstruct images successfully? While previous experiments used cropped images focusing on just the fish to train the autoencoder models, the entire frame is used here instead. The experiment applied the baseline model, with enhanced source images, and with the same settings as the previous experiment before the model training. After the model was trained, sample source images (both full scale and cropped), were tested against the autoencoder model to produce reconstructions.

**Figure 7:**
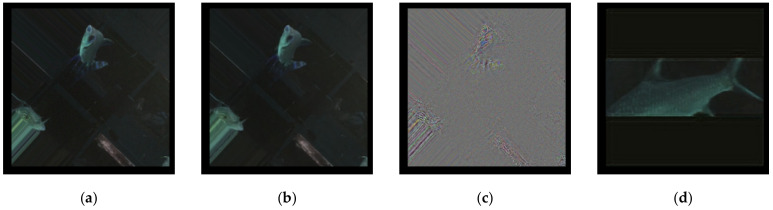
CNN full image autoencoder against cropped sample results: (**a**) source, (**b**) reconstructed, (**c**) difference, and (**d**) extended.

The model converged at the 189th epoch with fewer losses of (1.1481 × 10^−4^, 1.1045 × 10^−4^) than the baseline. The benchmark value was 0.2885. Figure 7 illustrates the results: Figure 7a is the randomly chosen full scale source image; Figure 7b is the resulting reconstructed image; Figure 7c is the standardized difference image between Figure 7a and Figure 7c; and Figure 7d is the reconstructed image of a cropped fish. The results show that an autoencoder model that was trained on full images can also reconstruct low-contrast images (Figure 7d) successfully, including even the particularly low-quality ones that were previously “unseen”.

**Figure 8:**
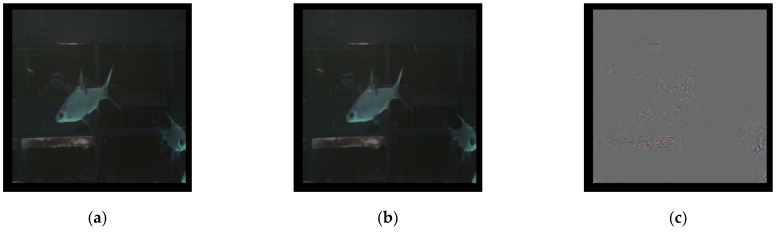
CNN cropped image autoencoder against full sample results: (**a**) source, (**b**) reconstructed, and (**c**) difference.

A new question then arises: can an autoencoder model trained from cropped images reconstruct full images? An autoencoder model trained only on cropped images should be missing training information when dealing with a full image, so how would the model reconstruct a full image? The results are illustrated in Figure 8; Figure 8a is the source full image, Figure 8b is the reconstructed image, and Figure 8c is the standardized difference image. The autoencoder can in fact successfully reconstruct a full image, despite seemingly lacking some information.

The answer is in the baseline model itself; through convolution and pooling, the CNN model can extract the image features required to reconstruct images regardless of the cropping. In conclusion, an autoencoder trained on just cropped images is sufficient to reconstruct a full image from a source. In other words, an aquarium could save computing resources by opting to train the autoencoder using cropped images over entire images.

## 7. Latent Space Database

The traditional relational database has a special feature: the binary large object (BLOB), which is able to store and to retrieve the images [28]. One drawback of using BLOB to retrieve the images is that the process needs a temporary buffer storage to hold the data. Another drawback is that the binary data needs to be serialized—the Base64 format is a common scheme—when transferring over the Internet. As with the BLOB, the character large object (CLOB) can store and retrieve the latent space in Base64 format as well. This transformation comes with a price of increasing the size of the data.

The aquatic subjects are identified and predicted by the object-detection neural model; the detected subjects are the input source buffer arrays to the autoencoder. These buffer arrays are dimensioned into the corresponding latent spaces; and then serialized and stored into the database. It is a continuous process—from video capture to the storing of the latent space—taking up sizeable computing resources and time for the task.

NoSQL is a promising solution to store and retrieve the latent spaces. Its robustness and redundancy can reduce the effort in backing up and maintaining the data within [29]. This paper stored the hourly video files under a file structure, the date, and twenty-four hours sub-folders. The metadata contains the information about where and when the video was recorded; along with the latent space data, a NoSQL database was used to store these data. The latent space size was $\left(32,\;32,\;64\right)$, 64 KB. After serialization in Base64 format, the size is increased up to 86 KB. Since an aquarium’s objective is to study the fishes’ behavior and habitation, the standardize difference images in binary form are adequate for such purposes. Many traditional relational databases cannot support the BLOB and CLOB exceeding 64 KB in size. Using NoSQL database to store and retrieve these tremendous number of latent spaces is inevitable [30].

## 8. Discussion

**Figure 9:**
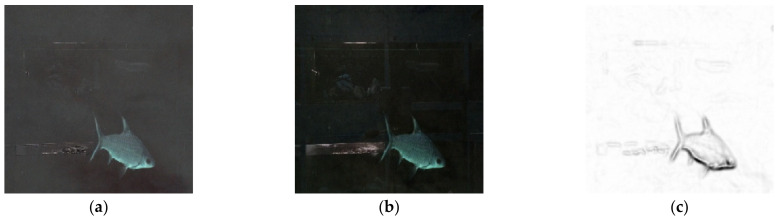
MLP auto-encoded images: (**a**) source image, (**b**) reconstructed image, and (**c**) Sobel treatment image.

This paper derives two different model structures, the MLP and the CNN. The experiments showed that applying the MLP model will result in better standardized difference images, which can replace the source low-contrast images; in addition, through a Sobel treatment for enhancing the object shapes [31] it will be handy to the aquarium operation in observing the aquatic subjects only, disregarding the background noises. Figure 9 illustrates the visual effects with MLP model (the dense layers of “128-64-32-64-128”, the training epochs were 16): Figure 9a shows the source image captured from the habitat; Figure 9b shows the reconstructed image by the retrained MLP autoencoder against the standardized difference images (clearer aquatic shape in visual); and Figure 9c shows the Figure 9b image after Sobel treatment; it is particularly useful in observing the aquatic subjects’ behavior, such as being ill or dying, in the habitat. The training loss versus the validated was (0.4157, 0.4171) after the 16th epoch. The model was slightly overfitting, but it was acceptable; the ones illustrated in Figure 9b,c have a practical use, especially when the caregiver has difficulty in observing the low-contrast images.

On the other hand, the CNN model (the convolution and max-pooling blocks are “128-64-32”, similar to the previous MLP model), has a better outcome in reconstructing the synthetic images, because the standardized differences are low—the difference between the source and the reconstructed one is less recognizable visually. The training loss versus the validated was (0.4075, 0.4086) after the 16th epoch; the model was slightly overfitting, but it was still acceptable. In practical use, the latent representatives are stored into the database to reconstruct the synthetic images instead of keeping those high-dimensional source images.

Using the color histogram method can disclose the color distribution and the frequencies about the colors used, respectively. Two approaches were taken: (1) applied and learnt the neural model against the FULL (covered entire compartment) images; and (2) the neural model trained on the OBJECT (covered the extracted boxed object only) images and applied the model against the FULL images. The validating samples contain the images that has not been seen by the training models.

The model performance was measured by the histogram matrices for the Standardized Difference (DIFF, the image differences of Source—Reconstructed) and DIFF2 (Sobel Treatment against DIFF) images. This paper calculated: (1) Model—the model structure ID; (2) Min.—the minimal value; (3) Max.—the maximal value; (4) Std. Dev.—the standard deviation of all histograms; (5) Scale—the subtraction of Max. and Min.; (6) COV.—the coefficient variance of all histograms; and (7) Image—the training dataset is either from FULL or OBJECT.

For MLP models (the symmetric layers of “64-32-16”, “128-64-32”, and “256-64”), the DIFF and DIFF2 images have the best visual outcomes for different purposes, not the reconstructed ones; therefore, the best performance model has the best distinguishability, e.g., the Train-Loss, Validate-Loss, COV., Std. Dev., and Scale values are the largest ones, respectively. The MLP models are applied to derive the Sobel images (DIFF2); therefore, this paper is only concerned about the loss values of the last epoch.

Table 4 illustrates the results and shows that the “256-64” model derived from OBJECT has the best performance which has the best distinguishability than others. However, considering the model training time, the FULL model training took approximate 365 ms/step, but the OBJECT models only took approximate 84 ms/step; therefore, this paper proposes the use the DIFF2 (Sobel treatment) derived from this OBJECT model to observe the aquatic object movements, because lots of background noises were greatly reduced.

**Figure 10:**
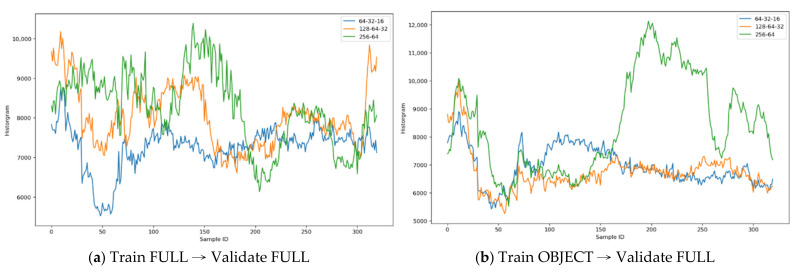
MLP model histograms comparison.

Figure 10 illustrates the histograms comparison across the MLP autoencoder models: Figure 10a—the autoencoder models trained on FULL images and validated on FULL images; other than the training dataset, it implies that the “256-64” has the best color distinguishability than others; Figure 10b—the autoencoder models trained on OBJECT images and validated on FULL images; it also implies that the “256-64” has the best color distinguishability than others.

For CNN models (the symmetric layers of “64-32-16”, “128-64-32”, “256-128-64-32”, and “512-256-128-64-32”), the reconstructed images are already good enough; the DIFF and DIFF2 histograms both have the most noises; therefore, the best performance model has the least distinguishability, e.g., the Validate-Loss, Accuracy, COV., Std. Dev., and Scale values are the smallest ones, respectively.

Table 5 illustrates the results and shows that the “512-256-128-64-32-64-126-256-512” models (both FULL and OBJECT) have similar distinguishability, validate-loss, and accuracy values, but considering the Accuracy and COV., this paper proposes to use the latent representatives derived from this FULL model to reconstruct the source images. Another finding worth noting is that using the OBJECT samples to reconstruct the source images does not always converge; these low-accuracy values explain that finding.

**Figure 11:**
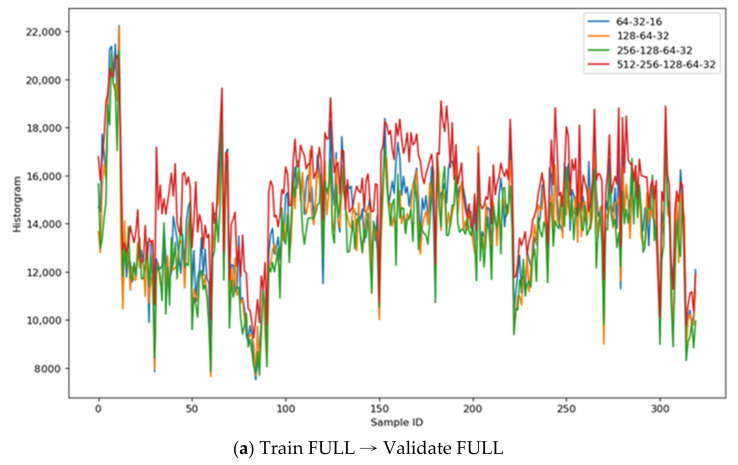

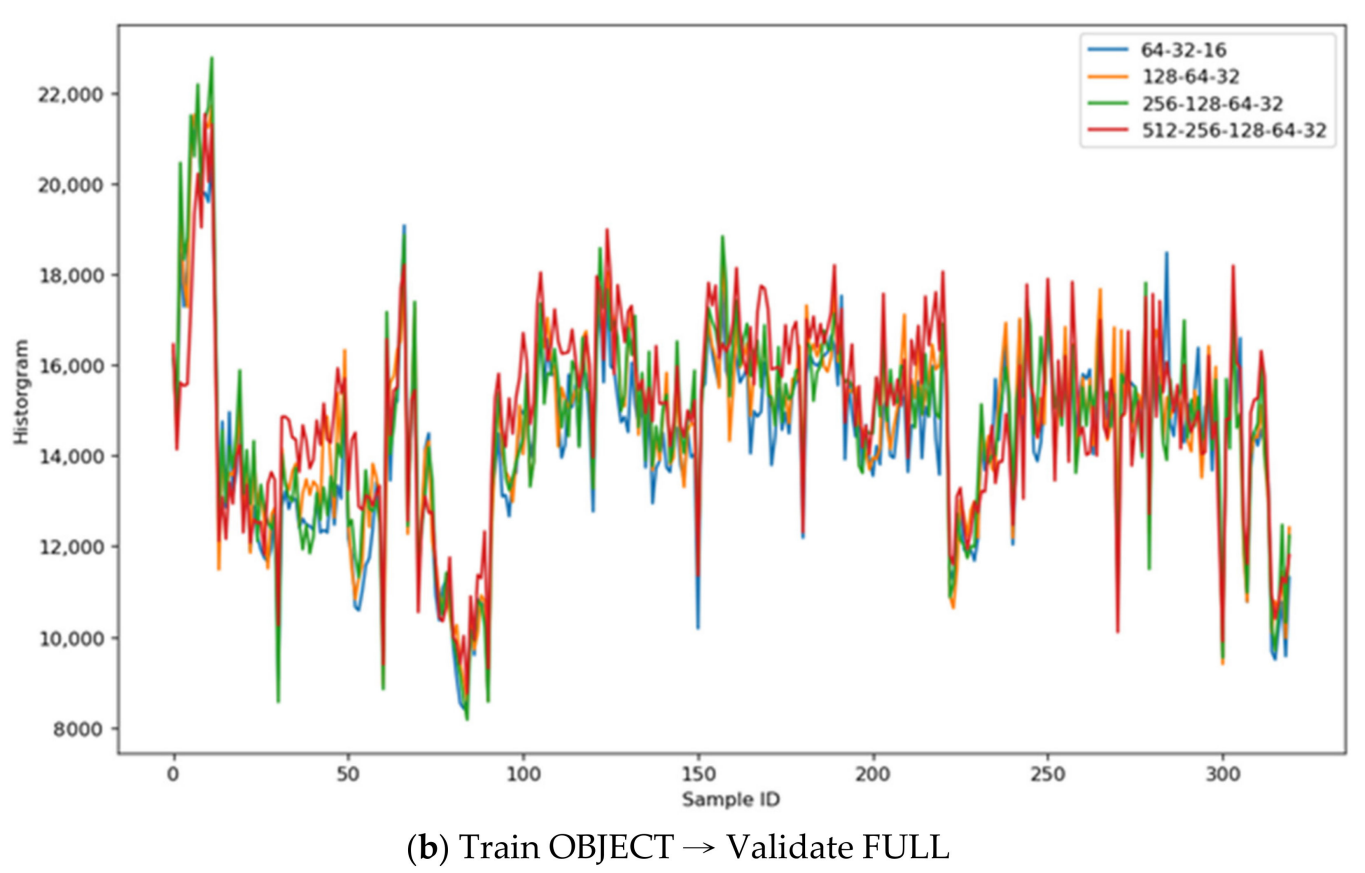
CNN model histograms comparison.

Figure 11 illustrates the histograms’ comparison across the CNN autoencoder models: Figure 11a—the autoencoder models trained on FULL images and validated on other FULL images; it implies that the “512-256-128-64-32” has the best color distinguishability than others; Figure 11b—the autoencoder models trained on OBJECT images and validated on FULL images; it also implies that the “512-256-128-64-32” has the best color distinguishability than others, but the training converged much earlier than the FULL model.

## 9. Conclusions

Just as with other neural networks, the model design of the autoencoder is data-driven, especially in image-related applications. The bottom line is that the model must serve a given purpose, which, in the context of an aquarium, is to store images of fish and other aquatic subjects filmed in their low-contrast environments effectively, with less dimensionality and yet retain the image fidelity to an acceptable degree with reduced noise and clearly defined shapes. This paper disclosed the insight of applying the autoencoder frameworks, by presenting the experiment results against the low-contrast images and concluded that both the MLP and the CNN have their appropriate uses in dealing with low-contrast image reconstruction. In addition, by applying the image preprocessing—enhancing the source images—it will make the autoencoder model reconstruct the images with more satisfactory results.

In a practical context, this paper concludes that a combination of both MLP and CNN approaches is better suited for an aquarium. This paper discovered that, through Equation (4) used during the MLP process, a standardized difference image was generated that satisfied the needs of an aquarium. The standardized difference image is not only sharp and well defined, but also removed unnecessary visual noise, retaining only relevant visual information pertaining to aquarium research and management. By using this difference image as a new source image for the CNN model, it provides an ideal solution for an aquarium’s architecture that requires the capability to handle complex configurations without excessive computing resources. To improve the low-contrast autoencoder models derived from the MLP frameworks, it may require a complex model structure that demands more computing resource than is available. For aquarium applications—the abnormality detection and behavior observation, “good enough”—considering the visual results, the dimensionality reduction, and the training time— is the rule of thumb, thus this paper does not seek to develop a superior autoencoder model than previous works, but is instead considered an implementation that is practical and feasible for aquarium operations.

## Figures and Tables

**Table 1:** Autoencoder model comparison with the previous works.

Authors	Framework	Data	Comparison
Ji et al. [15]	MLP	MNIST $\left(28,\;28,\;1\right)$ CIFAR10 $\left(32,\;32,\;3\right)$	Small dimensions High-contrast Simple model design
Betechuoh et al. [16]	MLP	HIV records	Estimation Function similar to the Equation (4)
Wang et al. [17]	CNN	BSDS500 $\left(481,\;321,\;1\right)$	Small dimensions High-contrast
Pihlgren et al. [18]	CNN	STL-10 $\left(96,\;96,\;3\right)$ SVHN $\left(32,\;32,\;3\right)$	Small dimensions High-contrast Simple model design
Dumas et al. [19]	CNN	BSDS300 $\left(481,\;321,\;1\right)$	Small dimensions High-contrast Image normalization preprocessing
Khan et al. [20]	CNN	Applied CICFlowMeter-V3 model to extract the cyberattack features from the CSE-CIC-IDS2018 dataset	Small dimensions Not an image source No image preprocessing required
Ramalho et al. [21]	CNN	The authors construct virtual scenes with multiple 3D views	Not an image source No image preprocessing required Generating synthetic 3D images

**Table 2:** MLP baseline training model. Bold indicates the latent layer.

Id	Layer	Type	Output Shape	Activation	Param #
IN	input	Input Layer	(None, 196,608)	ReLU	0
EN256	encoded_256	Dense	(None, 256)	ReLU	50,331,904
EN128	encoded_128	Dense	(None, 128)	ReLU	32,896
**L**	**latent**	**Dense**	**(None, 64)**	**ReLU**	**8256**
DE128	decoded_128	Dense	(None, 128)	ReLU	8320
DE256	decoded_256	Dense	(None, 256)	ReLU	33,024
OUT	output	Dense	(None, 196,608)	Sigmoid	50,528,256

**Table 3:** CNN baseline training model. Bold indicates the latent layer.

Id	Layer	Type	Output Shape	Activation	Param #
IN	input	Input Layer	(None, 256, 256, 3)	ReLU	0
CN_256	Conv2D_256	Conv2D	(None, 256, 256, 256)	ReLU	7168
MP_256	MaxPooling_256	MaxPooling2D	(None, 128, 128, 256)	ReLU	0
CN_128	Conv2D_128	Conv2D	(None, 128, 128, 128)	ReLU	295,040
MP_128	MaxPooling_128	MaxPooling2D	(None, 64, 64, 128)	ReLU	0
CN_64	Conv2D_64	Conv2D	(None, 64, 64, 64)	ReLU	73,792
**L**	**latent**	**MaxPooling2D**	**(None, 32, 32, 64)**	**ReLU**	**0**
CND_64	Conv2DT_64	Conv2DTranspose	(None, 32, 32, 64)	ReLU	36,928
US_256	UpSampling2D _64	UpSampling2D	(None, 64, 64, 64)	ReLU	0
CND_128	Conv2DT_128	Conv2DTranspose	(None, 64, 64, 128)	ReLU	73,856
US_128	UpSampling2D _128	UpSampling2D	(None, 128, 128, 128)	ReLU	0
CND_256	Conv2DT_256	Conv2DTranspose	(None, 128, 128, 256)	ReLU	295,168
US_256	UpSampling2D _256	UpSampling2D	(None, 256, 256, 256)	ReLU	0
OUT	output	Dense	(None, 256, 256, 3)	Sigmoid	6915

**Table 4:** MLP model performance against the standardized difference images.

Layers	Loss	Validate-Loss	Standard Deviation	Scale	Coefficient of Variance	Image
64-32-16	0.58	0.58	546.94	3213.96	0.08	F
	0.27	0.27	686.68	3474.33	0.10	O
128-64-32	0.58	0.58	764.69	3575.76	0.10	F
	0.27	0.27	736.14	4753.11	0.11	O
256-64	0.58	0.58	938.27	4253.11	0.11	F
	0.26	0.27	1719.05	6617.41	0.20	O

**Table 5:** CNN model performance against the standardized difference images.

Model	Loss	Accuracy	Standard Deviation	Scale	Coefficient of Variance	Image
64-32-16	0.56	84	2296.47	14,721.56	0.16	F
	0.26	83	2166.39	13,143.80	0.15	O
128-64-32	0.56	60	2196.73	14,540.64	0.16	F
	0.26	84	2206.08	13,135.36	0.15	O
256-128-64-32	0.56	86	2177.37	13,486.72	0.16	F
	0.26	84	2265.50	14,608.20	0.15	O
512-256-128-64-32	0.56	83	2163.02	11,761.30	0.14	F
	0.56	84	2131.25	12,779.87	0.14	O

## Data Availability

Available upon request from corresponding author.
